# Supplementary material for: Negative Acts in the Courtroom: Characteristics, Distribution, and Frequency among a National Cohort of Danish Prosecutors
Source: Behav Sci (Basel). 2024 Apr 16;14(4):332. doi: 10.3390/bs14040332 (PMC11047697; doi:10.3390/bs14040332)
Supplement: Supplementary file 1 [file behavsci-14-00332-s001.zip › behavsci-2931294-supplementary.pdf]

behavioral sciences

MDPI

Supplementary materials:

Geographical Region

Table S1 shows a statistically significant difference between the frequency of reported experiences with a lack of court management and discourtesy/dishonesty and the geographical area of employment. Prosecutors employed by the Eastern Danish Prosecution Service more frequently report experiencing illegitimate stressors due to a lack of court management compared to their colleagues in the Central and Western Danish Prosecution Services. Conversely, prosecutors employed by the Western Danish Prosecution Service report experiencing discourtesy/dishonesty more frequently than their colleagues in the Eastern and Central Danish Prosecution Services.

Table S1. Distribution of reported negative acts from professional counterparts across geographical areas of employment (the central, eastern, or western Prosecution Service) with *p*-values and adjusted standardized residuals from a Chi² test of independence.

Negative Acts	The Central Danish Prosecution Service			The Western Danish Prosecution Service			The Eastern Danish Prosecution Service			χ^2, p
	n	%	Adj. Std. R	n	%	Adj. Std. R	n	%	Adj. Std. R	
**Illegitimate tasks** *	<5	NA	−0.2	<5	NA	−1.8	15	4.5	1.8	3.730, $p = 0.145$
Illegitimate stressor										
**Lack of court management** *	**<5**	**NA**	**−0.8**	**<5**	**NA**	**−3.4**	**25**	**7.5**	**3.8**	**15.507, $p < 0.001$**
Exaggerated conflicts	9	9.0	1.6	11	4.5	−0.9	18	5.4	−0.2	2.780, $p = 0.249$
Illegitimate behavior										
Verbal abuse	15	15.0	−0.9	37	15.1	−1.5	71	21.3	2.1	4.456, $p = 0.108$
General incivility										
**Discourtesy/dishonesty**	**48**	**48.0**	**0.7**	**126**	**51.4**	**2.5**	**131**	**39.3**	**−2.9**	**8.766, $p = 0.012$**
Ignoring/exclusion/silencing *	6	6.0	1.8	5	2.0	−1.2	10	3.0	−0.1	3.728, $p = 0.170$
Professional discrediting	19	19.0	−0.4	50	20.4	−0.1	71	21.3	0.4	0.266, $p = 0.875$
Threats/intimidation	7	7.0	0.1	15	6.1	−0.5	24	7.2	0.4	0.271, $p = 0.873$
Person-focused incivility										
Gender/age disparagement	<5	NA	−0.6	15	6.1	0.7	17	5.1	−0.2	0.691, $p = 0.708$
Unprofessional address *	<5	NA	1.0	<5	NA	−0.4	<5	NA	−0.3	1.085, $p = 0.590$
Appearance comments *	<5	NA	0.8	<5	NA	0.1	<5	NA	−0.7	0.803, $p = 0.493$

Note: Adj. Std. R = adjusted standardized residuals; **bold** = values significant at $p < 0.05$; * = *p*-value from Fisher's exact test due to an expected cell count less than 5 (n < 5), and the corresponding NA for percentage refers to censured counts to maintain anonymity.
